# Preterm Neonatal Mortality and Its Associated Factors in Ethiopia: A Systematic Review and Meta‐Analysis

**DOI:** 10.1002/hsr2.72136

**Published:** 2026-03-23

**Authors:** Temesgen Gebeyehu Wondmeneh, Abdulhakim Hora Hedato

**Affiliations:** ^1^ Department of Public Health, College of Medical and Health Science Samara University Semera Ethiopia

**Keywords:** Ethiopia, mortality, neonate, preterm

## Abstract

**Background:**

Preterm neonatal mortality is the leading cause of death among children under 5 years of age globally, with the highest burden in low‐ and middle‐income countries. Evidence on the national pooled prevalence of preterm neonatal mortality and its determinants in Ethiopia is limited. This study aimed to estimate the pooled prevalence of preterm neonatal mortality and identify its associated factors in Ethiopia.

**Methods:**

A comprehensive search was carried out in Web of Science, Scopus, PubMed, Google Scholar, and African Journals Online. The Preferred Reporting Items for Systematic Reviews and Meta‐Analyzes (PRISMA) guidelines were followed to report the review findings. Data were extracted using a standardized Excel spreadsheet, and the quality of the included studies was assessed using the Newcastle‐Ottawa Scale. A random‐effects meta‐analysis model was used to estimate the pooled prevalence with a 95% confidence interval (CI). Heterogeneity across studies was assessed using I² statistic, and subgroup analyzes were performed to explore potential sources of heterogeneity.

**Results:**

Thirty‐one eligible articles were included in the analysis. The pooled prevalence of preterm neonatal mortality was 28.58% (95% CI: 23.08%–34.08%). Male sex (AHR = 1.26, 95% CI: 1.05–1.47), gestational age ≤ 32 weeks (AHR = 1.59, 95% CI: 1.02–2.16), lack of kangaroo mother care (AHR = 1.86, 95% CI: 1.05–2.67), asphyxia (AHR = 1.92, 95% CI: 1.48–2.35), a fifth‐minute APGAR score < 7 (AHR = 1.78, 95% CI: 1.25–2.32), respiratory distress (AHR = 1.69, 95% CI: 1.50–1.87), hypothermia (AHR = 1.28, 95% CI: 1.01–1.55), and sepsis (AOR = 1.60, 95% CI: 1.07–2.14) were identified as risk factors for preterm neonatal mortality. In contrast, antenatal care utilization (AHR = 0.65, 95% CI: 0.36–0.94) and antenatal steroid exposure (AHR = 0.65, 95% CI: 0.31–0.99) were protective factors against preterm neonatal mortality.

**Conclusion:**

More than one in four preterm neonates died in Ethiopia. Several neonatal and clinical factors, including male sex, very low gestational age of 32 weeks or less, lack of kangaroo mother care, asphyxia, a low fifth‐minute APGAR score, respiratory distress, hypothermia, and sepsis, were identified as significant predictors of preterm neonatal mortality and require timely intervention, appropriate medication, and close follow‐up. Conversely, antenatal care utilization and antenatal steroid exposure were found to be protective factors, highlighting the importance of quality maternal and newborn care services.

**Abbreviations**
AHR: adjusted hazard ratio
ANC: antenatal care
AOR: adjusted odd ratio
APGAR: appearance pulse grimace activity respiration
CI: confidence interval

## 1 Introduction

Preterm babies are those born alive before 37 completed weeks of gestation [1]. Globally, an estimated 13.4 million babies were born preterm in 2020 [2]. In Sub‐Saharan Africa, the prevalence of preterm birth ranged from 3.4% to 49.4% [3]. Complications of preterm birth are the leading cause of death among children under 5 years of age, accounting for approximately 900,000 deaths in 2019 [4]. Preterm neonates contributed to about 46% of all neonatal deaths, with 49% occurring in South Asia and 40% in Sub‐Saharan Africa [5]. In a study conducted in India and Pakistan, mortality rate of preterm neonates was 23.3% [6]. In East Africa, it was 473.6 per 1000 live births [7], while in Uganda, preterm neonatal mortality was reported at 19.8% [8]. In Ethiopia, the pooled prevalence of preterm neonatal death was 12.97% [9].

Several factors have been associated with preterm neonatal mortality. These include male sex, lack of antenatal care, gestational age under 32 weeks, respiratory distress syndrome, hypothermia, small for gestational age, and a fifth‐minute APGAR score below 7 [10, 11, 12]. Limited resources in neonatal intensive care units [13], birth asphyxia [14], lack of antenatal corticosteroid use [15], sepsis [16], and absence of kangaroo mother care [9] were also risk factors. Other contributors to preterm neonatal death include necrotizing enterocolitis [17], congenital malformations, and congenital infections [6, 16].

Estimating the burden of preterm neonatal mortality at the national level and identifying maternal and fetal factors that contribute to these deaths, especially in resource‐limited settings such as Ethiopia, is essential for developing effective interventions. Reliable data on the magnitude and determinants of preterm neonatal mortality can inform the design of targeted strategies and help prioritize limited resources for nationwide implementation. Although several studies have been conducted in different districts, there is a lack of comprehensive evidence summarizing the overall prevalence and key factors associated with preterm neonatal mortality in Ethiopia. Therefore, this systematic review and meta‐analysis aim to provide pooled estimates of preterm neonatal mortality and to identify its main determinants in the context of Ethiopia.

## 2 Methods

### 2.1 Reporting and Registration of Systematic Review and Meta‐Analysis

The results of this systematic review and meta‐analysis were reported in accordance with the Preferred Reporting Items for Systematic Reviews and Meta‐Analyzes (PRISMA) 2020 statement checklist [18] (Supporting Information S1). The protocol was registered in the International Prospective Register of Systematic Reviews (PROSPERO) under ID CRD42024524768 [19].

### 2.2 Search Strategies

Electronic databases, including Web of Science, Scopus, PubMed, Google Scholar, and African Journals Online, were systematically searched. In addition, reference lists were manually screened to ensure that no relevant articles were missed. The search terms were based on the following keywords: mortality, preterm neonate, and Ethiopia. In PubMed, “preterm neonate” and “mortality” were expanded using relevant synonyms and word variants in the advanced search. The search strategies were customized for each database and applied using appropriate Boolean operators (“AND” and “OR”). The initial search was conducted from April 28 to May 3, 2024, and was updated from February 11 to 15, 2026. The literature search was conducted carefully in various databases after a rigorous review and examination, with details of the 576 search strategies provided in the Supporting Information S2.

### 2.3 Operational Definition

Preterm neonate: defined as babies born alive before 37 weeks of pregnancy are completed [20].

### 2.4 Eligible Criteria (Inclusion and Exclusion)

The inclusion criteria were descriptive and observational studies that reported specific information on the prevalence of preterm neonatal mortality, studies conducted in Ethiopia, studies conducted on preterm neonates [20], and studies published in English. Qualitative studies, review articles, letters to the editor, case reports, and case series were excluded. When a study was published more than once (duplicate publication), the version with the largest sample size and the most detailed report was included.

### 2.5 Study Selection

EndNote software (X8.1) was used to remove duplicate records. Subsequently, the titles and abstracts of the identified articles were independently screened. The full texts of potentially eligible articles were then retrieved and assessed for eligibility, followed by full‐text screening for final inclusion. Agreement was ensured at each stage of the screening process, and any disagreements were resolved through scientific consensus.

### 2.6 Data Extraction

Data were extracted using a pretested data extraction form developed in Microsoft Excel. The extracted data were independently recorded and cross‐checked to ensure consistency. The following information was obtained from each included study: first author's name, year of publication, study region, study design, sample size, number of deaths among preterm neonates, and prevalence of mortality (Supporting Information S3).

### 2.7 Quality of Included Studies

The methodological quality of the included studies was assessed using an adapted version of the Newcastle–Ottawa Scale for cohort studies [21]. The tool evaluates three domains: selection, comparability, and outcome. The total score ranges from 0 to 9 and is categorized as follows: low risk of bias [7, 8, 9], moderate risk [5, 6], and high risk (0–4). Studies with moderate (scores 5–6) or low risk of bias (scores 7–9) were considered eligible for inclusion in the meta‐analysis. The methodological quality assessment was conducted independently, and any disagreements were resolved through scientific consensus.

### 2.8 Statistical Analysis

STATA software version 17 was used for the analysis. Heterogeneity across the studies was assessed using the I² statistic. I² values of 0%, 25%, 50%, and 75% were considered to indicate no, low, moderate, and high heterogeneity, respectively [22]. Due to significant heterogeneity among studies, a random‐effects meta‐analysis was performed to estimate the pooled prevalence with a 95% confidence interval (CI) [23]. Publication bias was assessed by visually inspecting the funnel plot, followed by Egger's test [24, 25]. A p‐value of less than 0.05 in the Egger's test was considered indicative of statistically significant publication bias. If publication bias was detected, a trim‐and‐fill analysis was performed. Subgroup analyzes were conducted to explore potential sources of heterogeneities among the studies. Sensitivity analyzes were performed to assess the robustness of the pooled prevalence estimate [26]. The pooled effects of adjusted hazard ratios (AHR) or adjusted odds ratios (AOR) were calculated using the AHRs or AORs of the contributing factors from the included studies to identify factors associated with preterm neonatal mortality. Finally, the results of the meta‐analysis were presented in forest plots and tables with a 95% confidence level.

### 2.9 Ethical Approval

Since the systematic review and meta‐analysis were used in previously published primary studies, ethical approval was not needed.

## 3 Results

### 3.1 Search Results

**Figure 1:**
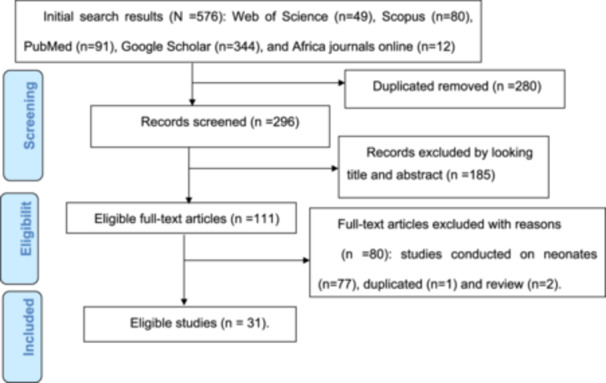
Shows the PRISMA flow chart for the selection of studies for systematic review.

A total of 576 articles were identified in the initial search from Web of Science, Scopus, PubMed, Google Scholar, and African Journals Online. After removing 280 duplicate articles using EndNote X8.1 software, 296 articles remained. During the title and abstract screening, 185 articles that were not related to the study objectives were excluded, leaving 111 articles for full‐text review. After reviewing the full texts, 80 articles were excluded for various reasons: 77 were not conducted on preterm neonates, one was a duplicate, and two were review articles. Finally, 31 eligible articles were included in this meta‐analysis (Figure 1).

### 3.2 Study Characteristics

Thirty‐one studies were included in this systematic review and meta‐analysis. Eight studies were conducted in the Amhara region, and six were conducted in Addis Ababa. Five studies were conducted in each of the Tigray, Oromia, and Southern Nations, Nationalities, and Peoples' (SNNP) regions. One study was conducted in Dire Dawa. Another study was conducted across multiple centers in different regions (Oromia, Amhara, and Addis Ababa) [27]. Twenty‐two studies were retrospective cohort studies, and the remaining nine were prospective cohort studies. The largest and smallest sample sizes were 5000 [28] and 197 [29], respectively. The minimum and maximum prevalence rates of preterm neonatal mortality were 4.2% [28] and 43.7% [30], respectively (Table 1).

**Table 1:** Included study characteristics.

Authors, publication years	Region	Study design	Sample size	Case (%)
Sinshaw; AE, et al. [31], 2019	Amhara	Retrospective	535	167 (31.2)
Huka et al. [32], 2023	Oromia	Retrospective	510	130 (25.5)
Tamene et al. [33], 2020	Amhara	Retrospective	686	247 (36.1)
Yismaw et al. [34], 2018	Amhara	Retrospective	516	149 (28.8)
Bereka et al. [35], 2021	SNNP	Retrospective	568	199 [35]
Girma et al. [36], 2021	Tigray	Retrospective	336	96 (28.6)
Girma et al. [37], 2023	Tigray	Retrospective	561	180 (32.1)
Tirore et al. [29], 2024	SNNP	Prospective	197	48 (24.4)
Birhanu et al. [38], 2022	AddisAbaba	Prospective	358	125 (34.9)
Abebaw et al. [39], 2021	Amhara	Retrospective	498	135 (27.11)
Gebreheat et al. [40], 2022	Tigray	Retrospective	1017	149 (14.6)
Hailemeskel et al. [41], 2023	Amhara	Prospective	456	132 (28.9)
Yehuala et al. [42], 2015	Amhara	Retrospective	485	122 (25.2)
Gebremeskel et al. [43], 2020	Tigray	Retrospective	346	77 (22.2)
Toma et al. [44], 2021	Oromia	Retrospective	505	127 (25.1)
Feleke et al.[45], 2022	SNNP	Retrospective	723	241 (33.3)
Genie et al. [46], 2022	Amhara	Retrospective	291	110 (37.8)
Aynalem et al. [47], 2020	AddisAbaba	Retrospective	571	170 (29.7)
Aynalem et al. [28], 2022	AddisAbaba	Retrospective	5000	210 (4.2)
Belay et al. [48], 2022	Amhara	Retrospective	542	167 (30.8)
Wesenu et al. [49], 2017	Oromia	Retrospective	490	171 (34.9%)
Mekasha et al. [27], 2020	N/A	Prospective	3773	1106 (29.3%)
Dagnachew et al. [50], 2019	AddisAbaba	Prospective	407	103 (25.3%)
Mihretie et al. [30], 2023	AddisAbaba	Prospective	277	121 (43.7%)
Mihretu et al. [51], 2024	SNNP	Prospective	614	200 (32.6%)
Mengesha et al. [52], 2025	Dire Dawa	Prospective	478	126 (26.4%)
Arersa et al. [53], 2025	Oromia	Retrospective	579	152 (26.3%)
Abera et al. [54], 2025	Oromia	Retrospective	259	42 (16%)
Tsega et al. [55], 2025	SNNP	Prospective	347	104 (30%)
Getaneh et al. [56], 2025	AddisAbaba	Retrospective	466	205 (43.9%)
Fisseha et al. [57], 2024	Tigray	Retrospective	480	109 (22.7%)

*Note*: N/A (not applicable) indicates no specific region (a study was conducted in different regions).

Abbreviation: SNNP, Southern Nation Nationality People.

### 3.3 Quality of Included Studies

The quality of included studies was assessed using the Newcastle–Ottawa Scale. Discrepancies in scoring were resolved through consensus. Of the 31 included studies, 29 received a score of 9/9, and the remaining 2 received a score of 8/9. All studies were classified as having a low risk of bias and were included in the final meta‐analysis (Supporting Information S4).

### 3.4 Pooled Prevalence of Preterm Neonatal Mortality

**Figure 2:**
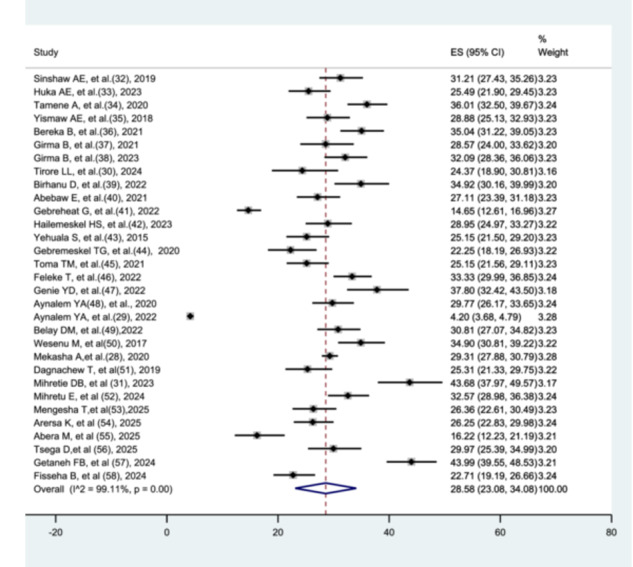
Pooled prevalence of preterm neonatal mortality.

In this systematic review and meta‐analysis, 22,871 preterm neonates were included, of whom 5420 died. Using a random‐effects meta‐analysis model, the pooled prevalence of preterm neonatal mortality was 28.58% (95% CI: 23.08–34.08), with substantial heterogeneity across studies (I² = 99.11%, *p* < 0.001) (Figure 2).

### 3.5 Subgroup Analysis

Subgroup analysis was performed by region, study design, and publication year to explore potential sources of heterogeneity in preterm neonatal mortality in Ethiopia. The highest pooled prevalence of preterm neonatal mortality was observed in SNNP (31.6%, 95% CI: 28.7%–34.6%), followed by Amhara (30.6%, 95% CI: 27.8%–33.4%) and Addis Ababa (30.2%, 95% CI: 13.0%–47.5%). Oromia and Dire Dawa reported pooled prevalence of 25.6% (95% CI: 20.4%–30.9%) and 26.4% (95% CI: 22.6%–30.5%), respectively, while Tigray had the lowest prevalence at 24.0% (95% CI: 17.0%–30.9%). Substantial heterogeneity was observed across most regions, with I² values ranging from 59.4% to 99.4%. Prospective studies showed a slightly higher pooled prevalence of preterm neonatal mortality (30.4%, 95% CI: 27.6%–33.2%) compared to retrospective studies (27.8%, 95% CI: 21.1%–34.5%). However, considerable heterogeneity was detected in both subgroups (I² = 79.5% and 99.1%, respectively; *p* < 0.001). Studies published between 2011 and 2020 reported a pooled prevalence of 29.3% (95% CI: 26.8%–31.8%), whereas those published between 2021 and 2025 showed a prevalence of 28.3% (95% CI: 21.6%–35.1%). High heterogeneity persisted in both periods (I² = 79.4% and 99.1%, respectively; *p* < 0.001) (Table 2).

**Table 2:** Subgroup analysis.

Variables	Categories	Prevalence (95%CI)	I^2^, p‐value	df
Region	Amhara	30.6% (27.8%–33.4%)	74.3%, < 0.001	7
	Oromia	25.6% (20.4%–30.9%)	88.8%, < 0.001	4
	SNNP	31.6% (28.7%–34.6%)	59.4%, 0.04	4
	Addis Ababa	30.2% (13%–47.5%)	99.4%, < 0.001	5
	Tigray	24.0% (17.0%–30.9%)	94.7%, < 0.001	4
	Dire Dawa	26.4% (22.6%–30.5%)		
Study design	Retrospective	27.8% (21.1%–34.5%)	99.1%, < 0.001	21
	Prospective	30.4% (27.6%–33.2%)	79.5%, < 0.001	8
Publication year	2011–2020	29.3% (26.8%–31.8%)	79.4%, < 0.001	8
	2021–2025	28.3% (21.6%–35.1%)	99.1%, < 0.001	21

Abbreviation: SNNP, Southern Nation Nationality People.

### 3.6 Publication Bias

**Figure 3:**
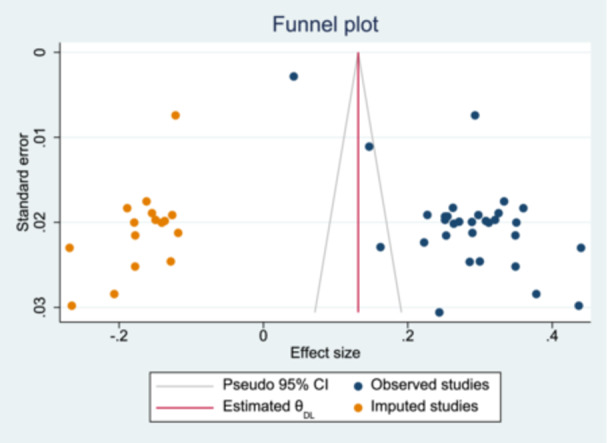
Funnel plots for observed and imputed studies.

Publication bias was assessed using funnel plot asymmetry and Egger's regression test. A funnel plot was constructed by plotting study‐specific prevalence estimates with their 95% confidence intervals against their standard errors. Visual inspection of the funnel plot suggested asymmetry, indicating possible publication bias in the pooled prevalence of preterm neonatal mortality. This finding was supported by Egger's regression test, which demonstrated statistically significant evidence of small‐study effects (*p* < 0.001). The intercept (bias coefficient) was significantly different from zero (β = 13.44, 95% CI: 10.54–16.33), indicating funnel plot asymmetry. To further evaluate the potential impact of publication bias, a trim‐and‐fill analysis was performed. The method identified 31 observed studies and estimated that 16 studies were potentially missing, which were imputed on the left side of the funnel plot. This suggests that studies with smaller effect sizes may be underrepresented. Before adjustment, the pooled effect size based on the observed studies was 0.286 (95% CI: 0.231–0.341). After imputing the potentially missing studies, the adjusted pooled effect size decreased substantially to 0.131 (95% CI: 0.078–0.184). This reduction suggests that the original pooled estimate may have been overestimated due to publication bias or small‐study effects (Figure 3).

### 3.7 Sensitivity Analysis

**Figure 4:**
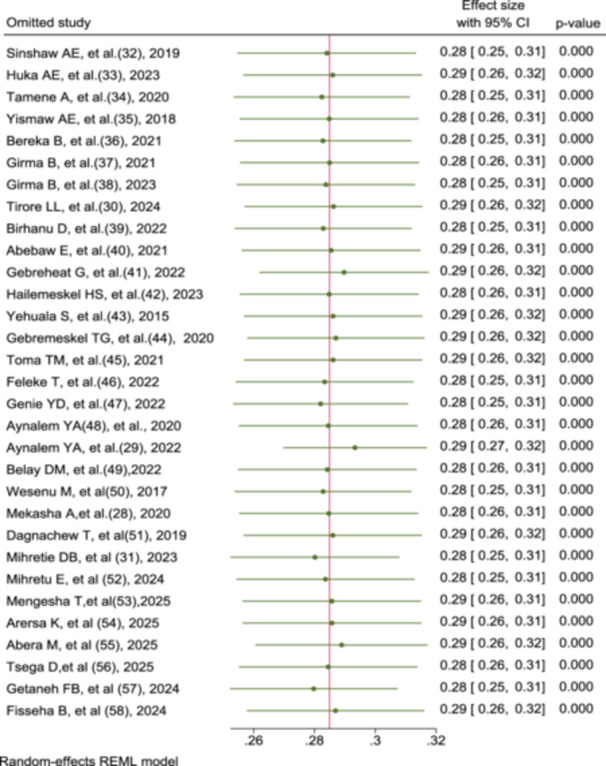
Sensitivity analysis to determine the effect of a single study on the overall pooled prevalence of preterm neonatal mortality.

The leave‐out‐one sensitivity analysis was conducted to assess the influence of each individual study on the overall pooled estimate of preterm neonatal mortality. The results show that when each study was sequentially omitted, the pooled effect size ranged narrowly between 0.28 and 0.29, with overlapping 95% confidence intervals. This minimal variation in the pooled estimates indicates that no single study had a disproportionate influence on the overall result. The pooled prevalence remained stable regardless of which study was excluded, suggesting that the meta‐analysis findings are robust and reliable (Figure 4).

### 3.8 Determinants of Preterm Neonatal Mortality

#### 3.8.1 The Association Between Gender and Preterm Neonatal Mortality

**Figure 5:**
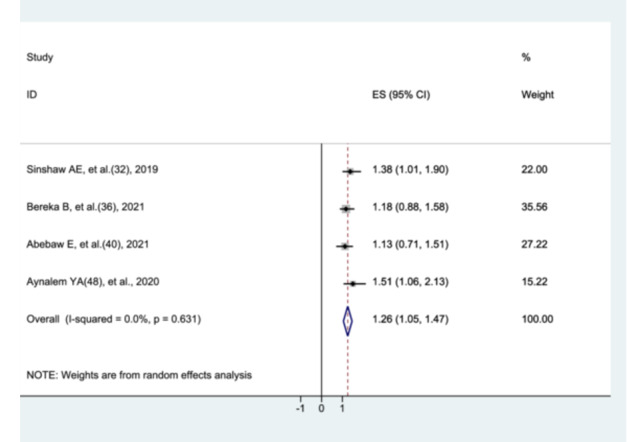
The association between male gender and preterm neonatal mortality.

Four studies [31, 35, 39, 47]. examined the association between male sex and preterm neonatal mortality. The meta‐analysis showed that male preterm neonates had a significantly higher risk of mortality compared with female preterm neonates (AHR = 1.26; 95% CI: 1.05–1.47), with no evidence of heterogeneity among included studies (Figure 5).

#### 3.8.2 The Association Between ANC Utilization and Preterm Neonatal Mortality

**Figure 6:**
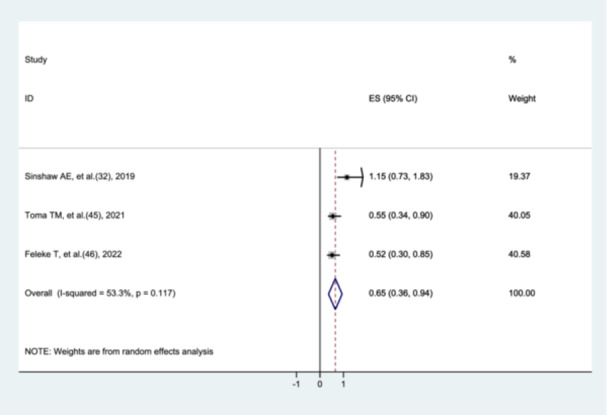
The association between ANC utilization and preterm neonatal mortality.

To assess the association between antenatal care (ANC) utilization and preterm neonatal mortality, three studies [31, 44, 45] were included. The meta‐analysis showed that preterm neonates born to mothers who utilized ANC had a 35% lower risk of mortality compared with those whose mothers did not utilize ANC (AHR = 0.65, 95% CI: 0.36–0.94). There was moderate heterogeneity among the included studies (I² = 53.3%, *p* = 0.117) (Figure 6).

#### 3.8.3 The Association Between Preeclampsia and Preterm Neonatal Mortality

**Figure 7:**
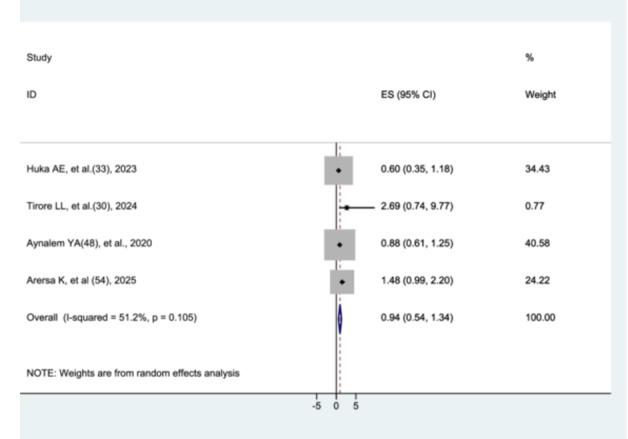
The association between preeclampsia and preterm neonatal mortality.

Four studies were included to examine the association between preeclampsia and preterm neonatal mortality [29, 32, 47, 53]. The results of the current meta‐analysis showed that there was no statistically significant difference in preterm neonatal mortality between neonates born to mothers with preeclampsia and those without preeclampsia (AHR = 0.94, 95% CI: 0.55–1.34). No significant heterogeneity (*p* = 0.105) was observed among the included studies (Figure 7).

##### 3.8.3.1 The Association Between Gestational Age and Preterm Neonatal Mortality

**Figure 8:**
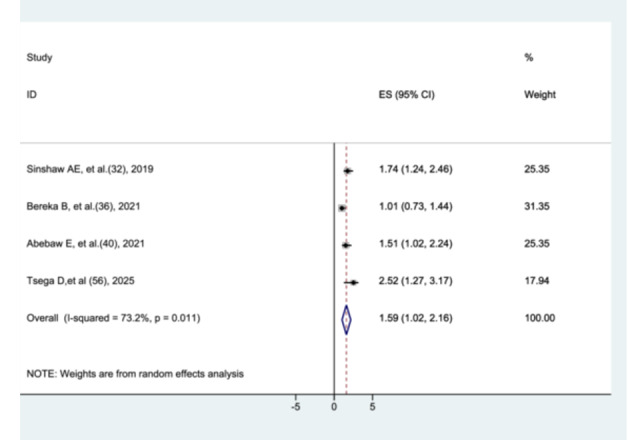
The association between gestational age and preterm neonatal mortality.

To assess the association between gestational age and preterm neonatal mortality, four studies [31, 35, 39, 55] were included. In four of these studies, gestational age was categorized into two groups: ≤ 32 weeks and > 32 weeks. This meta‐analysis found that preterm neonates with a gestational age of ≤ 32 weeks had a significantly higher risk of mortality compared to those with a gestational age of > 32 weeks (AHR = 1.59, 95% CI: 1.02–2.16). Significant heterogeneity was observed among the studies (I² = 73.2%, *p* = 0.011) (Figure 8).

#### 3.8.4 The Association Between Birth Weight and Preterm Neonatal Mortality

**Figure 9:**
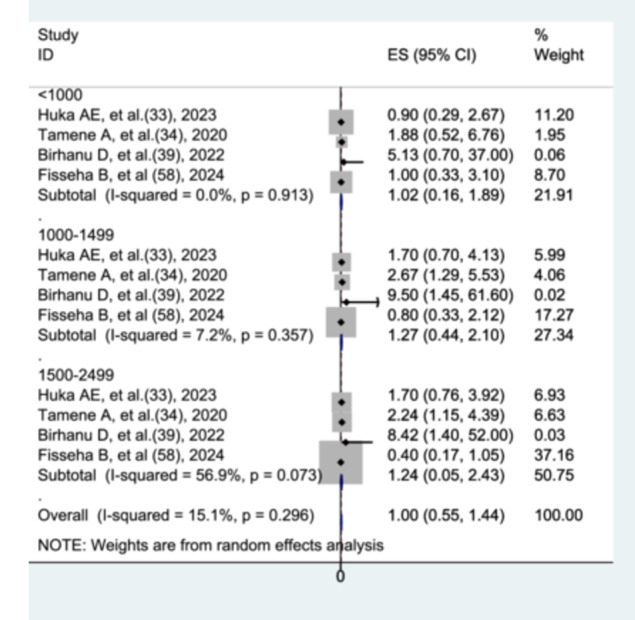
The association between birth weight and preterm neonatal mortality.

To evaluate the association between birth weight and preterm neonatal mortality, four studies [32, 33, 38, 57] were included in the analysis. Across these studies, birth weight was categorized into four groups: < 1000 g, 1000–1499 g, 1500–2499 g, and ≥ 2500 g (reference group). Compared with preterm neonates weighing ≥ 2500 g, there was no statistically significant difference in mortality risk among those with a birth weight of < 1000 g (AHR = 1.02; 95% CI: 0.16–1.89), 1000–1499 g (AHR = 1.27; 95% CI: 0.44–2.10), or 1500–2499 g (AHR = 1.24; 95% CI: 0.05–2.43). No heterogeneity was observed among studies assessing birth weight < 1000 g, low heterogeneity was found for those assessing 1000–1499 g, and significant heterogeneity was observed among studies evaluating birth weight 1500–2499 g (Figure 9).

#### 3.8.5 The Association Between Kangaroo Mother Care and Preterm Neonatal Mortality

**Figure 10:**
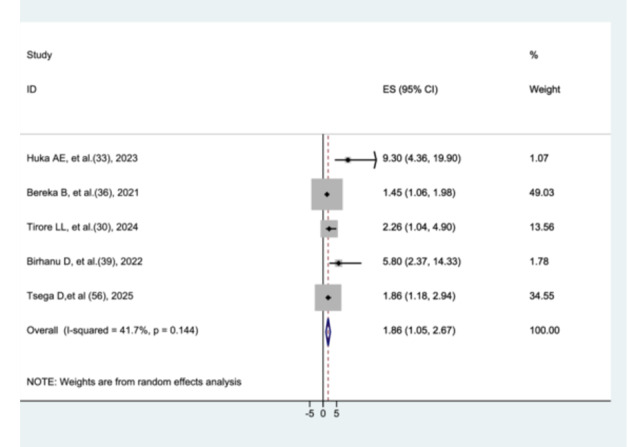
The association between kangaroo method care and preterm neonatal mortality.

The association between kangaroo mother care and preterm neonatal mortality was assessed using five studies [29, 32, 35, 38, 55]. Preterm neonates who did not receive kangaroo mother care had a significantly higher risk of mortality compared to those who received kangaroo mother care (AHR = 1.86, 95% CI: 1.05–2.67). Moderate heterogeneity was observed among the included studies (I² = 41.7%, *p* = 0.144) (Figure 10).

#### 3.8.6 The Association Between Birth Asphyxia and Preterm Neonatal Mortality

**Figure 11:**
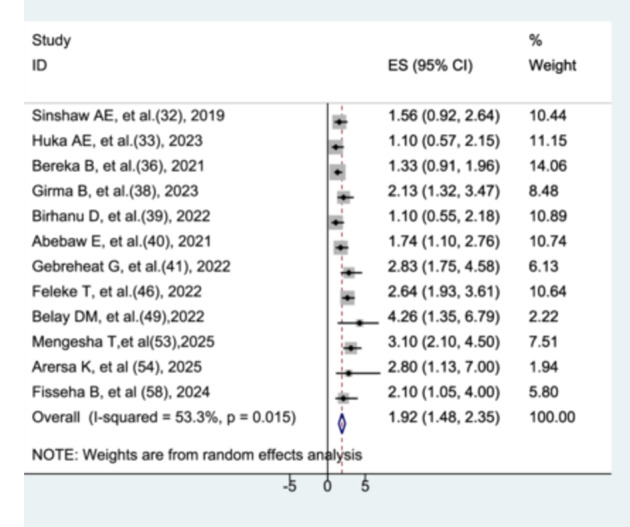
The association between birth asphyxia and preterm neonatal mortality.

The association between birth asphyxia and preterm neonatal mortality was evaluated using twelve studies [31, [32], [35], [37], [38], [39], [40], [45], [48], [52], [53], 57]. Preterm neonates with birth asphyxia had a 1.92‐fold higher risk of mortality compared to those without birth asphyxia (AHR = 1.92, 95% CI: 1.48–2.35). Moderate heterogeneity was observed among the included studies, and this was statistically significant (I² = 53.3%, *p* = 0.015). (Figure 11).

#### 3.8.7 The Association Between APGAR Score and Preterm Neonatal Mortality

**Figure 12:**
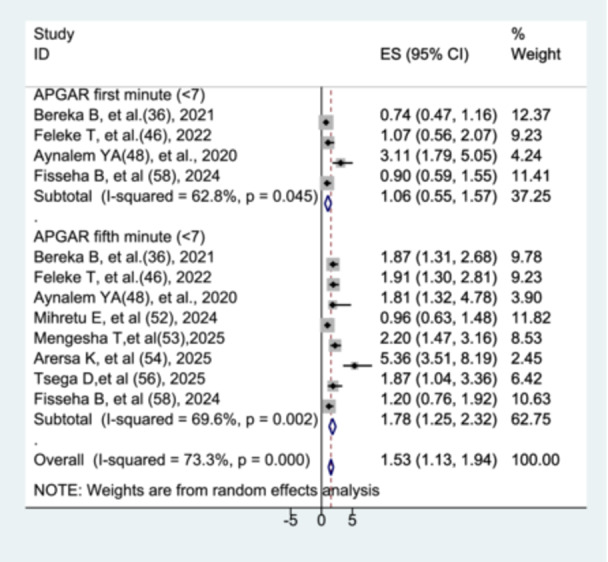
The association between APGAR score and preterm neonatal mortality.

The association between APGAR score and preterm neonatal mortality was evaluated separately for the first and fifth minutes. Four studies [35, 45, 47, 57] assessed the relationship for the first‐minute APGAR score, while eight studies [35, 45, 47, 51, 52, 53, 55, 57] assessed the fifth‐minute APGAR score. Preterm neonates with a first‐minute APGAR score less than 7 did not have a significantly different mortality rate compared to those with a score of 7 or higher (AHR = 1.06; 95% CI: 0.55–1.57), with heterogeneity among studies (I² = 62.8%, *p* = 0.045). In contrast, preterm neonates with a fifth‐minute APGAR score less than 7 had a significantly higher risk of mortality, nearly 1.8 times that of neonates with a score of 7 or higher (AHR = 1.78; 95% CI: 1.25–2.32), with substantial heterogeneity observed among studies (I² = 69.6%, *p* = 0.002) (Figure 12).

#### 3.8.8 The Association Between Respiratory Distress Syndrome and Preterm Neonatal Mortality

**Figure 13:**
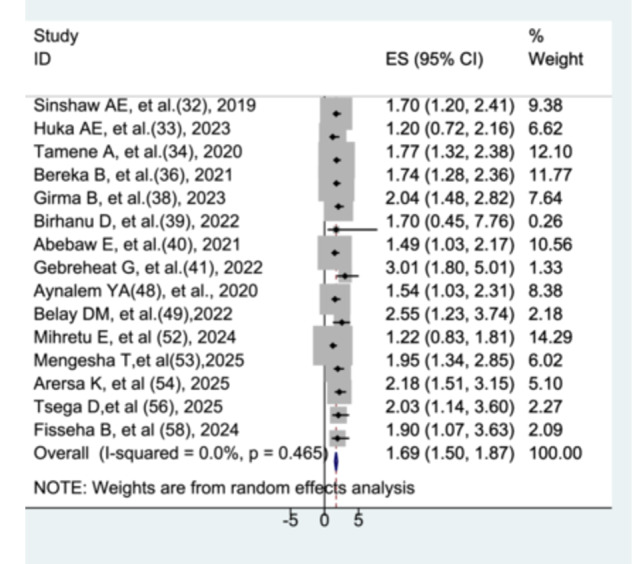
The association between respiratory distress syndrome and preterm neonatal mortality.

Fifteen studies [31, 32, 33, 35, 37, 38, 39, 40, 47, 48, 51, 52, 53, 55, 57] were included to assess the association between respiratory distress syndrome and preterm neonatal mortality. The meta‐analysis indicated that preterm neonates with respiratory distress syndrome had a 1.69 times higher risk of mortality compared to those without respiratory distress (AHR = 1.69, 95% CI: 1.50–1.87), with no significant heterogeneity observed among the studies (Figure 13).

#### 3.8.9 The Association Between Hypoglycemia and Preterm Neonatal Mortality

**Figure 14:**
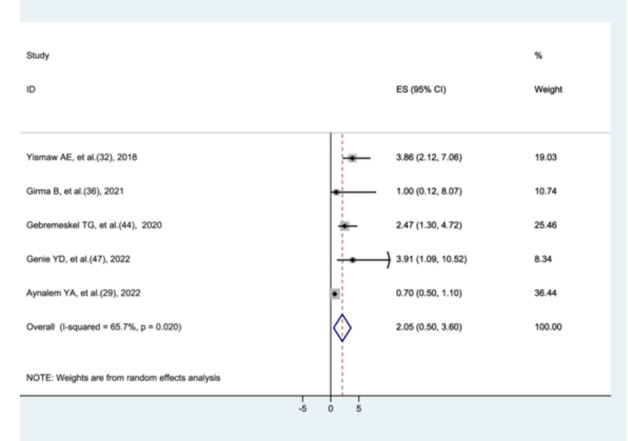
The association between hypoglycemia and preterm neonatal mortality.

The association between hypoglycemia and preterm neonatal mortality was evaluated in five studies [28, 31, 35, 43, 46]. The meta‐analysis showed that preterm neonates with hypoglycemia did not have a significantly higher mortality rate compared to those without hypoglycemia (AOR = 2.05, 95% CI: 0.5–3.6). Heterogeneity was observed among the studies (I² = 65.7%, *p* = 0.02) (Figure 14).

#### 3.8.10 The Association Between Hypothermia and Preterm Neonatal Mortality

**Figure 15:**
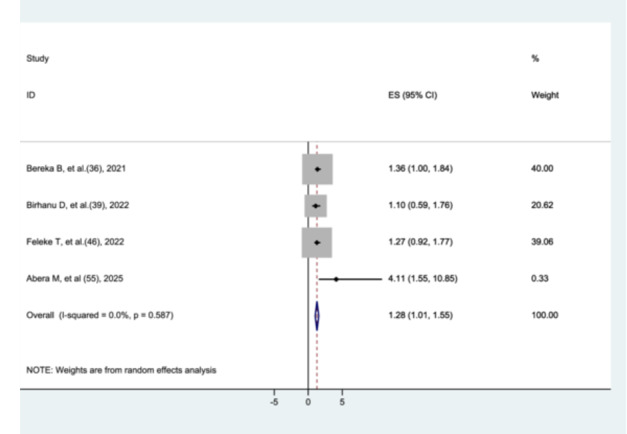
The association between hypothermia and preterm neonatal mortality.

The effect of hypothermia on preterm neonatal mortality was evaluated in four studies [35, 38, 45, 54]. The results indicated that preterm neonates with hypothermia had a modest but statistically significant increase in mortality compared to those without hypothermia (AHR = 1.28, 95% CI: 1.01–1.55) (Figure 15).

#### 3.8.11 The Association Between Premature Rupture of Membrane and Preterm Neonatal Mortality

**Figure 16:**
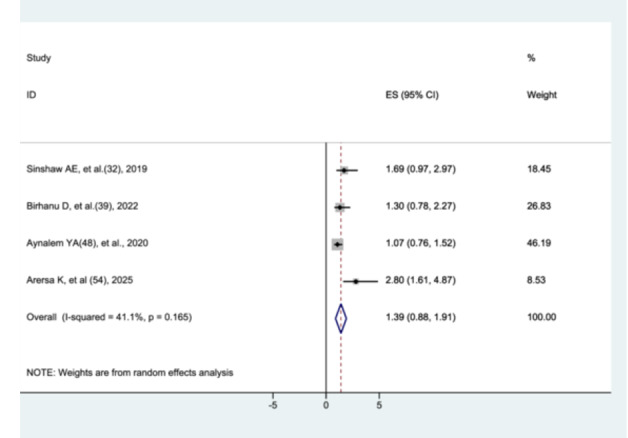
The association between premature rupture of membrane and preterm neonatal mortality.

The association between premature rupture of membranes and preterm neonatal mortality was assessed in four studies [31, 38, 47, 53]. The meta‐analysis showed that there was no significant difference in mortality between preterm neonates with premature rupture of membranes and those without (AHR = 1.39, 95% CI: 0.88–1.91) (Figure 16).

#### 3.8.12 The Association Between Sepsis and Preterm Neonatal Mortality

**Figure 17:**
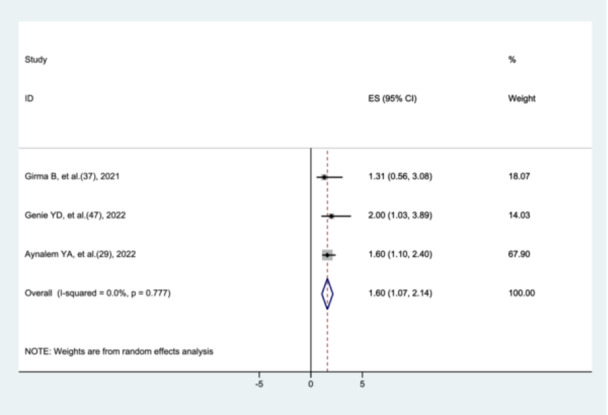
The association between sepsis and preterm neonatal mortality.

Three studies [28, 36, 46] were included to assess the association between sepsis and preterm neonatal mortality. The results of this systematic review and meta‐analysis showed that preterm neonates with sepsis were more likely to die than those without sepsis (AOR = 1.6, 95% CI: 1.07–2.14) (Figure 17).

#### 3.8.13 Association Between Antenatal Steroid Exposure and Preterm Neonatal Mortality

**Figure 18:**
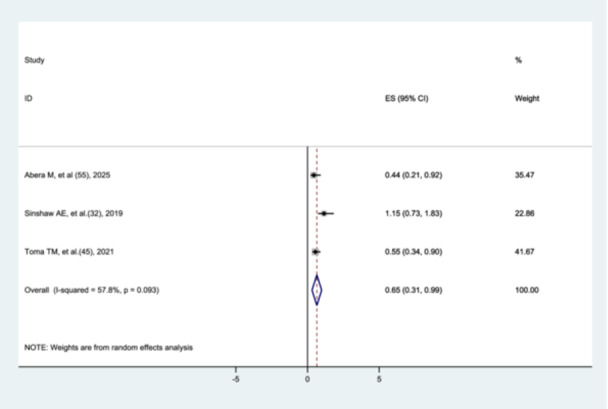
Association between antenatal steroid exposure and preterm neonatal mortality.

Three studies [31, 44, 54] were included to assess the association between antenatal steroid exposure and preterm neonatal mortality. The findings of the meta‐analysis showed that preterm neonates with a history of antenatal steroid exposure were less likely to die than those without such exposure (AHR = 0.65, 95% CI: 0.31–0.99) (Figure 18).

## 4 Discussion

This systematic review and meta‐analysis estimated that the pooled prevalence of preterm neonatal mortality in Ethiopia was 28.58% (95% CI: 23.08%–34.08%), indicating a substantial burden among preterm neonates. This prevalence is higher than previous pooled estimates from Ethiopia (12.97%) [9], as well as reports from India and Pakistan (23.3%) [6] and Uganda (19.8%) [8]. The observed differences may be explained by variations in study settings, populations, and methodological approaches. The present review included a large number of hospital‐based studies, many conducted in neonatal intensive care units, where more critically ill preterm neonates are admitted. As noted in previous literature, limited neonatal intensive care resources and variability in service availability in low‐resource settings can increase the risk of mortality among preterm neonates [13]. Although the pooled prevalence of preterm neonatal mortality in Ethiopia (28.6%) is substantial, it is lower than some reported estimates for East Africa [7] and Sub‐Saharan Africa [3, 5]. This discrepancy may be due to differences in study populations, including congenital malformations and infections [16, 17], variations in healthcare access [10, 15], and differences in neonatal care practices. Nevertheless, the findings remain consistent with global evidence showing that complications of preterm birth are a leading cause of under‐five mortality [4].

Regional subgroup analysis showed variation in preterm neonatal mortality across Ethiopian regions, with the highest prevalence observed in SNNP and the lowest in Tigray. These differences may reflect variations in access to maternal and neonatal health services, quality of neonatal intensive care, and socioeconomic conditions. Previous studies have suggested that disparities in health service availability and neonatal care capacity contribute to differences in neonatal survival across settings [10, 13, 15]. The substantial heterogeneity observed across regions and study designs further indicates differences in study populations, health system capacity, and clinical management practices. Prospective studies reported slightly higher mortality than retrospective studies, which may be attributed to better identification and documentation of deaths in prospective designs. Retrospective studies often rely on medical records that may be incomplete or subject to reporting bias, potentially leading to underestimation of mortality. Similarly, the relatively stable prevalence across publication periods suggests that, despite improvements in maternal and newborn care, preterm neonatal mortality remains a persistent public health problem in Ethiopia.

This review identified several predictors of preterm neonatal mortality. Male preterm neonates had a higher risk of death than females, which is consistent with previous studies [10, 11, 12]. This difference may be related to biological vulnerability and delayed lung maturation among male neonates, which increases susceptibility to complications [58]. Gestational age ≤ 32 weeks were also associated with increased mortality, consistent with earlier findings [10, 11, 12]. Extremely preterm neonates are more likely to experience physiological immaturity and complications that increase the risk of death [59]. Lack of kangaroo mother care was a significant predictor of mortality, which agrees with previous evidence [9]. Kangaroo mother care improves thermal regulation, breastfeeding, and infection prevention, thereby enhancing survival among preterm neonates [60]. Similarly, respiratory distress and hypothermia were associated with increased mortality, consistent with previous studies [10, 11, 12]. These conditions reflect the physiological immaturity of preterm neonates and the challenges of maintaining adequate thermal and respiratory support in resource‐limited settings [61]. Birth asphyxia and a low fifth‐minute APGAR score were important predictors of mortality, consistent with earlier reports [14] and previous studies identifying low APGAR scores as a risk factor [10, 11, 12]. Birth asphyxia results from impaired blood flow and oxygen delivery to the fetus immediately before, during, or after birth. This oxygen deprivation can lead to hypoxic‐ischemic injury, brain cell death, and, in severe cases, neonatal death [62]. Sepsis was another significant predictor of mortality, in agreement with previous evidence [16], highlighting the substantial contribution of infections to preterm neonatal deaths. Sepsis can trigger immune dysregulation, including lymphocyte apoptosis, which contributes to immunosuppression and increases the risk of mortality [63]. In addition, poor infection prevention practices and delayed diagnosis and treatment may further elevate the risk of adverse outcomes. Antenatal care utilization was associated with reduced mortality, consistent with previous studies identifying lack of antenatal care as a risk factor [10, 11, 12]. Antenatal care provides opportunities for early detection and management of pregnancy complications and preparation for preterm birth, thereby improving neonatal outcomes [64]. Similarly, antenatal steroid exposure was protective, supporting previous findings that the absence of antenatal corticosteroid use increases mortality risk [15]. Antenatal corticosteroids promote fetal lung maturation and reduce complications associated with prematurity, ultimately enhancing survival [65].

Most of the included studies were retrospective and relied on medical records, which may have been incomplete. Consequently, some important predictors of preterm neonatal mortality, such as maternal sociodemographic characteristics, nutritional status, and institutional factors, were not consistently reported. In addition, the hospital‐based nature of these retrospective studies may have introduced selection bias, as preterm neonates who died at home were not included; therefore, the findings should be interpreted with caution when generalizing to the wider population. Substantial heterogeneity was observed among the included studies, even after subgroup analyzes. This heterogeneity may be attributed to methodological differences across studies, variations in healthcare settings, and differences in study populations. These include neonatal factors (e.g., congenital anomalies), maternal factors (e.g., obstetric complications), and health system factors such as inequalities in infection prevention practices, shortages of essential equipment, limited skilled personnel, and restricted access to healthcare facilities. Evidence of publication bias and small‐study effects was detected, and trim‐and‐fill analysis suggested that the pooled prevalence may have been overestimated. This may reflect the limited availability or dissemination of research conducted in resource‐constrained settings. In addition, some regions were underrepresented, which may affect the representativeness of the findings. Finally, because all included studies were observational, causal relationships cannot be established, and residual confounding may remain. Variations in study design, regional representation, and outcome measurement may also influence the precision and generalizability of the pooled estimates.

However, this study has several important strengths. A comprehensive and up‐to‐date literature search was conducted across multiple databases, complemented by manual screening of reference lists to ensure broad coverage of relevant studies. Study selection, data extraction, and quality assessment were independently performed by two reviewers, which minimized potential bias and improved the reliability of the review. Most of the included studies were conducted after 2020, allowing the findings to reflect the recent burden of preterm neonatal mortality in the country. In addition, this study provides a precise national estimate of preterm neonatal mortality and comprehensively identifies preventable risk factors. Subgroup analyzes were performed to explore potential sources of heterogeneity, while leave‐one‐out sensitivity analysis demonstrated stable pooled estimates, indicating the robustness of the findings.

## 5 Conclusion

More than one in four preterm neonates in Ethiopia dies during the neonatal period, indicating a persistently high mortality rate. Key predictors include male sex, very low gestational age (≤ 32 weeks), absence of kangaroo mother care, asphyxia, low fifth‐minute APGAR score (< 7), respiratory distress, hypothermia, and sepsis. Preterm neonates with these conditions require breathing support, appropriate medication, and close follow‐up. In contrast, antenatal care (ANC) utilization and antenatal steroid exposure were protective factors, underscoring the importance of improving access to quality maternal and newborn care services to reduce preterm neonatal mortality. Collaborative efforts by national, regional, and local stakeholders are essential to address these challenges.

## Author Contributions

**Temesgen Gebeyehu Wondmeneh and Abdulhakim Hora Hedato:** conceptualization, investigation, funding acquisition, writing – original draft, methodology, validation, visualization, writing – review and editing, software, formal analysis, project administration, data curation, supervision, resources.

## Funding

The authors received no specific funding for this work.

## Ethics Statement

The authors have nothing to report.

## Conflicts of Interest

The authors declare no conflicts of interest.

## Transparency Statement

The lead author Temesgen Gebeyehu Wondmeneh affirms that this article is an honest, accurate, and transparent account of the study being reported; that no important aspects of the study have been omitted; and that any discrepancies from the study as planned (and, if relevant, registered) have been explained.

## Supporting information


**S1 File:** Checklist of Preferred Reporting Items for Systematic Reviews and Meta‐Analysis (PRISMA 2020).

**S2 File:** Comprehensive search strategy for preterm neonatal mortality in Ethiopia.

**S3 File:** Extracted data for preterm neonatal mortality in Ethiopia.

**S4 File:** Quality assessment of included studies.

## Data Availability

All data generated or analyzed during this study are included in this article.
