# Supplementary material for: Preterm Neonatal Mortality and Its Associated Factors in Ethiopia: A Systematic Review and Meta‐Analysis
Source: Health Sci Rep. 2026 Mar 23;9(3):e72136. doi: 10.1002/hsr2.72136 (PMC13097469; doi:10.1002/hsr2.72136)
Supplement: Supplementary file 2 — S2 File: Comprehensive search strategy for preterm neonatal mortality in Ethiopia. [file HSR2-9-e72136-s003.docx]

S2 File. Comprehensive search strategy for preterm neonatal mortality in Ethiopia

| Databases | Key search terms or phrases |
| --- | --- |
| **Web of science** | "Preterm neonates" [Exact phrase] Mortality [Subject] Ethiopia [Title] |
| Limited by | articles and English language |
| Total articles | 49 |
| **Scopus** | "Preterm neonate" mortality Ethiopia |
| Limited by | research articles and open access |
| Total articles | 80 |
| **PubMed** | (((((((((((((Mortality[MeSH Terms]) OR (Mortality)) OR (Mortalities)) OR ("Mortality Rate")) OR ("Mortality Rates")) OR ("Rate, Mortality")) OR ("Death Rate")) OR ("Death Rates")) OR ("Rate, Death")) OR ("Mortality Determinants")) OR ("Mortality Determinant")) OR ("Determinants Mortality")) AND (((((((((("Premature Birth"[MeSH Terms]) OR ("Premature Birth")) OR ("Birth, Premature")) OR ("Births, Premature")) OR ("Premature Births")) OR ("Preterm Birth")) OR ("Birth, Preterm")) OR ("Births, Preterm")) OR ("Preterm Births")) OR ("Preterm neonates"))) AND ((Ethiopia[MeSH Terms]) OR (Ethiopia)) |
| Limited by | free full text, human and English language |
| Total articles | 91 |
| **Google scholar** | Mortality OR death "preterm neonate" Ethiopia |
| Total articles | 344 |
| **African Journals Online** | “Preterm neonates” mortality Ethiopia |
| Total articles | 12 |
| **Total articles retrieved from all** **databases** | **576** |
