# Supplementary material for: Preterm Neonatal Mortality and Its Associated Factors in Ethiopia: A Systematic Review and Meta‐Analysis
Source: Health Sci Rep. 2026 Mar 23;9(3):e72136. doi: 10.1002/hsr2.72136 (PMC13097469; doi:10.1002/hsr2.72136)
Supplement: Supplementary file 3 — S3 File: Extracted data for preterm neonatal mortality in Ethiopia. [file HSR2-9-e72136-s001.docx]

S3 File Extracted data for preterm neonatal mortality in Ethiopia

| **ID** | **Authors, publication years** | **Region** | **Study design** | **Sample Size** | Case (%) | **prevalence** |
| --- | --- | --- | --- | --- | --- | --- |
|  | Sinshaw AE, et al.(32), 2019 | Amhara | Retrospective | 535 | 167 | 31.20% |
|  | Huka AE, et al.(33), 2023 | Oromia | Retrospective | 510 | 130 | 25.50% |
|  | Tamene A, et al.(34), 2020 | Amhara | Retrospective | 686 | 247 | 36.10% |
|  | Yismaw AE, et al.(35), 2018 | Amhara | Retrospective | 516 | 149 | 28.80% |
|  | Bereka B, et al.(36), 2021 | SNNP | Retrospective | 568 | 199 | 35% |
|  | Girma B, et al.(37), 2021 | Tigray | Retrospective | 336 | 96 | 28.60% |
|  | Girma B, et al.(38), 2023 | Tigray | Retrospective | 561 | 180 | 32.10% |
|  | Tirore LL, et al.(30), 2024 | SNNP | Prospective | 197 | 48 | 24.40% |
|  | Birhanu D, et al.(39), 2022 | Addis Ababa | Prospective | 358 | 125 | 34.90% |
|  | Abebaw E, et al.(40), 2021 | Amhara | Retrospective | 498 | 135 | 27.11% |
|  | Gebreheat G, et al.(41), 2022 | Tigray | Retrospective | 1017 | 149 | 14.60% |
|  | Hailemeskel HS, et al.(42), 2023 | Amhara | Prospective | 456 | 132 | 28.90% |
|  | Yehuala S, et al.(43), 2015 | Amhara | Retrospective | 485 | 122 | 25.20% |
|  | Gebremeskel TG, et al.(44),2020 | Tigray | Retrospective | 346 | 77 | 22.20% |
|  | Toma TM, et al.(45), 2021 | Oromia | Retrospective | 505 | 127 | 25.10% |
|  | Feleke T, et al.(46), 2022 | Sidama | Retrospective | 723 | 241 | 33.30% |
|  | Genie YD, et al.(47), 2022 | Amhara | Retrospective | 291 | 110 | 37.80% |
|  | Aynalem YA(48), et al.,2020 | Addis Ababa | Retrospective | 571 | 170 | 29.70% |
|  | Aynalem YA, et al.(29), 2022 | Addis Ababa | Retrospective | 5000 | 210 | 4.20% |
|  | Belay DM, et al.(49),2022 | Amhara | Retrospective | 542 | 167 | 30.80% |
|  | Wesenu M, et al (50), 2017 | Oromia | Retrospective | 490 | 171 | 34.9% |
|  | Mekasha A,et al.(28), 2020 | N/A | Prospective | 3773 | 1106 | 29.31% |
|  | Dagnachew T, et al(51), 2019 | Addis Ababa | Prospective | 407 | 103 | 25.3% |
|  | Mihretie DB, et al (31), 2023 | Addis Ababa | Prospective | 277 | 121 | 43.7% |
|  | Mihretu E, et al (52), 2024 | SNNP | Prospective | 614 | 200 | 32.6% |
|  | Mengesha T,et al(53),2025 | Dire Dawa | Prospective | 478 | 126 | 26.4% |
|  | Arersa K, et al (54), 2025 | Oromia | Retrospective | 579 | 152 | 26.3% |
|  | Abera M, et al (55), 2025 | Oromia | Retrospective | 259 | 42 | 16% |
|  | Tsega D,et al (56), 2025 | SNNP | Prospective | 347 | 104 | 30% |
|  | Getaneh FB, et al (57), 2025 | Addis Ababa | Retrospective | 466 | 205 | 43.9% |
|  | Fisseha B, et al (58), 2024 | Tigray | Retrospective | 480 | 109 | 22.7% |

N/A (not applicable) indicates no specific region (a study was conducted in different regions).

SNNP: Southern Nation Nationality People
