# Supplementary material for: Preterm Neonatal Mortality and Its Associated Factors in Ethiopia: A Systematic Review and Meta‐Analysis
Source: Health Sci Rep. 2026 Mar 23;9(3):e72136. doi: 10.1002/hsr2.72136 (PMC13097469; doi:10.1002/hsr2.72136)
Supplement: Supplementary file 4 — S4 File: Quality assessment of included studies. [file HSR2-9-e72136-s004.docx]

S4 File The quality of included studies using the Newcastle-Ottawa Scale Appraisal Checklist for cohort studies for preterm neonatal mortality

| Author, Publication year | Selection | | | | Comparability | Outcome | | | Total score out of 9  (%) | Risk of Bias |
| --- | --- | --- | --- | --- | --- | --- | --- | --- | --- | --- |
|  | 1 | 2 | 3 | 4 |  | 1 | 2 | 3 |  |  |
| Sinshaw AE, et al.(32), 2019 | 1 | 1 | 1 | 1 | 2 | 1 | 1 | 1 | 9/9 (100) | Low |
| Huka AE, et al.(33), 2023 | 1 | 1 | 1 | 1 | 2 | 1 | 1 | 1 | 9/9 (100) | Low |
| Tamene A, et al.(34), 2020 | 1 | 1 | 1 | 1 | 2 | 1 | 1 | 1 | 9/9 (100) | Low |
| Yismaw AE, et al.(35), 2018 | 1 | 1 | 1 | 1 | 2 | 1 | 1 | 1 | 9/9 (100) | Low |
| Bereka B, et al.(36), 2021 | 1 | 1 | 1 | 1 | 2 | 1 | 1 | 1 | 9/9 (100) | Low |
| Girma B, et al.(37), 2021 | 1 | 1 | 1 | 1 | 2 | 1 | 1 | 1 | 9/9 (100) | Low |
| Girma B, et al.(38), 2023 | 1 | 1 | 1 | 1 | 2 | 1 | 1 | 1 | 9/9 (100) | Low |
| Tirore LL, et al.(30), 2024 | 0 | 1 | 1 | 1 | 2 | 1 | 1 | 1 | 8/9 (88.9) | low |
| Birhanu D, et al.(39), 2022 | 1 | 1 | 1 | 1 | 2 | 1 | 1 | 1 | 9/9 (100) | Low |
| Abebaw E, et al.(40), 2021 | 1 | 1 | 1 | 1 | 2 | 1 | 1 | 1 | 9/9 (100) | Low |
| Gebreheat G, et al.(41), 2022 | 1 | 1 | 1 | 1 | 2 | 1 | 1 | 1 | 9/9 (100) | Low |
| Hailemeskel HS, et al.(42), 2023 | 1 | 1 | 1 | 1 | 2 | 1 | 1 | 1 | 9/9 (100) | Low |
| Yehuala S, et al.(43), 2015 | 1 | 1 | 1 | 1 | 2 | 1 | 1 | 1 | 9/9 (100) | Low |
| Gebremeskel TG, et al.(44),2020 | 1 | 1 | 1 | 1 | 2 | 1 | 1 | 1 | 9/9 (100) | Low |
| Toma TM, et al.(45), 2021 | 1 | 1 | 1 | 1 | 2 | 1 | 1 | 1 | 9/9 (100) | Low |
| Feleke T, et al.(46), 2022 | 1 | 1 | 1 | 1 | 2 | 1 | 1 | 1 | 9/9 (100) | Low |
| Genie YD, et al.(47), 2022 | 1 | 1 | 1 | 1 | 2 | 1 | 1 | 1 | 9/9 (100) | Low |
| Aynalem YA(48), et al.,2020 | 1 | 1 | 1 | 1 | 2 | 1 | 1 | 1 | 9/9 (100) | Low |
| Aynalem YA, et al.(29), 2022 | 1 | 1 | 1 | 1 | 2 | 1 | 1 | 1 | 9/9 (100) | Low |
| Belay DM, et al.(49),2022 | 1 | 1 | 1 | 1 | 2 | 1 | 1 | 1 | 9/9 (100) | Low |
| Wesenu M, et al (50), 2017 | 1 | 1 | 1 | 1 | 2 | 1 | 1 | 1 | 9/9 (100) | Low |
| Mekasha A,et al.(28), 2020 | 1 | 1 | 1 | 1 | 2 | 1 | 1 | 1 | 9/9 (100) | Low |
| Dagnachew T, et al(51), 2019 | 1 | 1 | 1 | 1 | 2 | 1 | 1 | 1 | 9/9 (100) | Low |
| Mihretie DB, et al (31), 2023 | 0 | 1 | 1 | 1 | 2 | 1 | 1 | 1 | 8/9 (100) | Low |
| Mihretu E, et al (52), 2024 | 1 | 1 | 1 | 1 | 2 | 1 | 1 | 1 | 9/9 (100) | Low |
| Mengesha T,et al(53),2025 | 1 | 1 | 1 | 1 | 2 | 1 | 1 | 1 | 9/9 (100) | Low |
| Arersa K, et al (54), 2025 | 1 | 1 | 1 | 1 | 2 | 1 | 1 | 1 | 9/9 (100) | Low |
| Abera M, et al (55), 2025 | 1 | 1 | 1 | 1 | 2 | 1 | 1 | 1 | 9/9 (100) | Low |
| Tsega D,et al (56), 2025 | 1 | 1 | 1 | 1 | 2 | 1 | 1 | 1 | 9/9 (100) | Low |
| Getaneh FB, et al (57), 2025 | 1 | 1 | 1 | 1 | 2 | 1 | 1 | 1 | 9/9 (100) | Low |
| Fisseha B, et al (58), 2024 | 1 | 1 | 1 | 1 | 2 | 1 | 1 | 1 | 9/9 (100) | Low |

Low risk (7-9), moderate risk (5–6), and high risk (0–4)

**Descriptions**

| **Assessment of quality of a cohort study – Newcastle Ottawa Scale** |  |
| --- | --- |
| 1. **Selection (4 points),** (**NB exposure = intervention**) |  |
| 1. Representativeness of the intervention cohorta) Truly representative of the average, elderly, community-dwelling resident ★b) Somewhat representative of the average, elderly, community-dwelling resident ★c) Selected group of patients, e.g. only certain socio-economic groups/areasd) No description of the derivation of the cohort | 🞏  🞏  🞏  🞏 |
| 2. Selection of the non-intervention cohorta) Drawn from the same community as the intervention cohort ★b) Drawn from a different sourcec) No description of the derivation of the non-intervention cohort | 🞏  🞏  🞏 |
| 3. Ascertainment of interventiona) Secure record (e.g. health care record) ★b) Structured interview ★c) Written self-reportd) Other / no description | 🞏  🞏  🞏  🞏 |
| 4. Demonstration that outcome of interest was not present at start of studya) Yes ★b) No | 🞏  🞏 |
| Comparability (maximum 2 points) |  |
| 1. Comparability of cohorts on the basis of the design or analysisa) Study controls for age, sex, marital status ★b) Study controls for any additional factors (e.g. socio-economic status, education) ★ | 🞏  🞏 |
| Outcome (3 points) |  |
| 1. Assessment of outcomea) Independent blind assessment ★b) Record linkage ★c) Self-report d) Other / no description | 🞏  🞏  🞏  🞏 |
| 2. Was follow up long enough for outcomes to occura) Yes, if median duration of follow-up >= 6 month ★b) No, if median duration of follow-up < 6 months | 🞏  🞏 |
| 3. Adequacy of follow up of cohortsa) Complete follow up: all subjects accounted for ★b) Subjects lost to follow up unlikely to introduce bias: number lost <= 20%, ★ or description of those lost suggesting no different from those followedc) Follow up rate < 80% (select an adequate %) and no description of those lostd) No statement | 🞏  🞏  🞏  🞏 |
